# When citizen science meets radon building diagnosis: Synthesis of a French pilot project developed in the framework of the European RadoNorm research project

**DOI:** 10.12688/openreseurope.15968.2

**Published:** 2023-08-08

**Authors:** Sylvain Andresz, Ambre Marchand-Moury, Joëlle Goyette-Pernot, Anne-Laure Rivière, Caroline Schieber

**Affiliations:** 1 Nuclear Protection Evaluation Centre (CEPN), Fontenay-aux-Roses, 92260, France; 2 Centre for Studies and Expertise on Risks, the Environment, Mobility and Urban Planning (Cerema), Autun, 71400, France; 3 Transform Institute, Romand Centre for Indoor Air Quality and Radon (croqAIR); School of Engineering and Architecture of Fribourg (HEIA-FR), HES-SO University of Applied Sciences and Arts of Western Switzerland, Fribourg, 1700, Switzerland; 4 Pays Vesoul Val-de-Saône, Vesoul, 70007, France

**Keywords:** Radon at home, RadoNorm, citizen science, radiation protection, radon diagnosis, ethics

## Abstract

As part of the European RadoNorm research project, citizen science pilot projects focusing on the management of radon risk in houses have been implemented in four countries. This article describes the methodological basis, the development and the results of the French pilot project. Building on an initial review of existing literature, the pilot project aims to frame a ‘participatory approach’ aligned with the standards and recognized practices of citizen science. Particular attention was given to the management of data and the inclusion of ethical considerations.

The focal point of the project was the process of radon building diagnosis which is supposed to be carried out whenever (high) radon concentrations are measured and should be prerequisite to mitigation works. As experience shows, however, this diagnosis is hardly implemented in France. To help remedy this situation, the pilot project recruited citizens already aware about radon from Pays Vesoul Val-de-Saône (East of France) to test an existing online self-evaluation guide for radon diagnosis, report on their operational experience and meet with radon/building experts. This enabled citizens to contribute to improvements in form and content to the guide and to ensure that it would be better fit for purpose. Comparison of the guide with experts’ practices offered additional perspectives on what building diagnosis should entail.

The pilot project produced rich and high-quality data that will nurture the evolution of the guide. The project demonstrated both the viability and the utility of applying the citizen science approach to radon post-measurement phases, with measurable benefits in bridging knowledge gaps and in encouraging behavioural changes. The results of using a citizen science approach in the field of radon management and research are encouraging, and they far outweigh the challenges involved in the implementation.

## Plain language summary

RadoNorm is a research project funded by the European Commission. Among its panel of research activities, one is dedicated to the testing of citizen science pilot projects on the the management of radon in houses. A pilot-project was developed in summer 2022 in France.

Radon is a naturally occurring radioactive gas created from some geological formations, it can travel to the surface, infiltrate buildings and accumulate. Radon exposure increases the risk of lung cancer. After an measurement showing (elevated) radon concentration in a house, a diagnosis (identify where the radon comes from) and mitigation (concrete actions to reduce radon) should follow, but evidence indicate that these are rarely performed in France. The authors thought it might be appropriate to focus the pilot project on the diagnosis step. From the point of view of citizen science, the project aimed to align with the recognized principles and practices in citizen science, include ethical considerations.

A pre-exiting on-line radon diagnosis guide was submitted to citizens recruited in the Pays de Vesoul (East of France) already aware about radon. The suggestions from the citizens were collected through questionnaires, meetings with radon/building experts and tests in real conditions. This ‘participatory approach’, positioning the citizen at the core of the project, produced technical suggestions that could help to improve the guide and, hopefully, increase diagnosis/mitigation intention.

Specific issues when merging citizen science and radon were identified (for example: a potential low participation and attention to plan the protection of the participants against radon from the onset) but these are manageable and should not prevent the launch other citizen science projects, which bring interesting and unexplored potentials for the citizens – and the experts – in radon management at home.

## Introduction

### Citizen science pilot projects in RadoNorm

Radon ( ^222^Rn) is a radioactive gas produced by the decay of uranium and radium naturally present in the Earth's crust. Transported from the soil to the surface, radon can enter into buildings through cracks, holes and porous materials and accumulate indoors, thus presenting a health risk. Epidemiological studies have presented evidence of a linear increases in relative lifetime risk of lung cancer at ≥ 100 Bq/m ^3^ of repetitive, long term radon exposure ( Darby *et al*., 2005; Krewski *et al*., 2005). Radon is recognized in many countries as the second cause of lung cancer deaths ( IARC, 1998; WHO, 2009).

RadoNorm is a Euratom Horizon 2020 research project aiming to strengthen the scientific and technical basis of the management of exposure situations to radon and other naturally occurring radioactive materials (NORM). From 2020 to 2025, RadoNorm will federate 57 organizations including universities, research centres, radiation protection institutes, etc., under a multidisciplinary approach bringing together research, social science, technological development, education, and training activities, all structure into eight Work Packages (WP) ( Kulka *et al.*, 2022).

One of the objectives of WP6 (‘Social Aspects’) is to investigate the perspectives offered by citizen science for the management of indoor radon in houses. Citizen science pilot projects were developed and tested in four countries: France (the pilot project being coordinated by the Nuclear Protection Evaluation Centre, CEPN), Ireland (by the Environmental Protection Agency, EPA), Hungary (by the Atomic Energy Research Centre, EK-CER) and Norway (by the Norwegian University for Life Sciences, NMBU). Each pilot project has its own specificities and organizational arrangements ( Martell *et al.*, 2022).

The purpose of this article is to describe the methodological tenets of the French pilot project, to present how the project unfolded during the summer 2022, and to discuss the results obtained, both in technical terms and with respect to citizen science.

### What is citizen science?

WP6 partners published a critical review of the literature on citizen science applied to radon research published until 2020 ( Martell *et al.*, 2021). The first observation made by Martell *et al*. is that 'citizen science' is a flexible concept whose boundaries have evolved over time and as a function of the various topics, disciplines and promoters involved. These research dynamics have made it difficult to produce a single and definitive definition of “citizen science”. Broadly, the term refers to any form of production of scientific knowledge where citizens actively and deliberately participate along with the researchers ( MESR, 2016). Yet even this broad definition fails to encompass the variety of methods applied in different disciplines ( Haklay *et al.*, 2021; Heigl *et al.*, 2019). This situation has prompted several researchers to define distinct categories of citizen science. Among these, Martell *et al*. proposed to adopt Hacklay's typology ( Haklay, 2013) based on the level of participation of citizens in the project ( Table 1).

**Table 1.** Four levels of participation in citizen science (adapted from Haklay, 2013).

Level	Denomination	Role of the citizens
**Level 1**	Crowdsourcing	Citizens as sensors
**Level 2**	Distributed intelligence	Citizens contribute with data and help with basic interpretation
**Level 3**	Participatory science	Citizens participate in problem definition, data collection, and drafting of the conclusion and reports
**Level 4**	Extreme citizen science	Citizens are deeply engaged in most parts, including data analysis, conclusion and broadcasting the results

Citizen science has been employed in a variety of research fields, especially biodiversity, environmental research and natural sciences ( MESR, 2016). The literature discussing its advantages and drawbacks is abundant. The most frequently mentioned advantages of citizen science include the volume of data collected and analysed, a favourable cost-time ratio, the development of innovative protocols ( *ibid*.; Heigl *et al.*, 2019), an increase in awareness/education, and encouraging social innovation ( *grassroots initiative*) ( Butkevičienė *et al.*, 2021). But citizen science also has significant downfalls:

- From the researcher’s perspective, citizen science challenges the principle of research autonomy, produces lower quality data, and may be exposed to an overall lack of rigor ( Elliott & Rosenberg, 2019);
- From the perspective of the citizens, there are risks of instrumentalization, discrepancy between the answers provided by the project and actual needs, unbalanced expert-citizen relationships, and unsustainable motivation ( Eleta *et al.*, 2019; MESR, 2016).

The management of data provided/produced by citizens as well as the implementation of ethical principles have been the topics of recent literature ( Heigl & Dörler, 2018; Oberle *et al.*, 2019), including a special issue of *Citizen Science, Theory and Practice* entirely dedicated to ethics ( Rasmussen & Cooper, 2019).

Several organizations offer recommendations and guidance on citizen science implementation. Martell *et al.* have adopted the 'Ten Principles of Citizen Science' developed by the European Citizen Science Association ( ECSA, 2015; ECSA, 2020) as a metric to judge whether a participatory initiative can be considered citizen science, and if adequate resources and best practices have been included in the project.

### State of the art on citizen science projects applied to radon management and research

The number of papers published on citizen science projects applied to radon management and research has been modest: less than 10 projects in the range 1984 to October 2020 have been identified by Martell *et al*. The role of the citizens has been generally low (‘level 1’ under Hacklay’s typology) and none has met the Ten Principles fully. Martell *et al*. highlighted that these projects followed a top-down approach, with a clear separation of tasks between experts and citizens, the latter acting as ‘data collectors’ and not involved in the design of the project, the production of scientific knowledge or in the development of the findings. The authors showed that earlier projects focused only on the first steps of radon management – namely, information about radon and measurement at home – while the post-measurement stages focusing on reducing radon exposure were not tackled.

These results should be adjusted in considering recent publications. There have been several studies on citizen science applied to radon published since 2020 and some addressed post measurement stages, such as reaction to radon test results ( Irvine *et al*., 2022) and the effect of building codes and practices on radon concentration ( Khan *et al*., 2021). The effect of citizen scientists’ psychology and behavioral choices with residential radon exposure (therefore providing means to reduce it) were also investigated recently ( Cholowsky *et al*., 2023; Simms *et al*., 2021).

### Objectives of the French pilot project

In France, no regulation regarding the management of radon in dwellings is currently in force ^1^. For about 20 years, local stakeholders such as municipalities, communities/counties (groups of municipalities), Regional Health Agencies (ARS) and associations have decided on a voluntary basis to develop radon awareness initiatives and implement measurement campaigns ( DGS, 2018). The Local Health Contract (CLS), which establishes a health strategy between a given community and the ARS, is sometimes an instrument used to formalize and implement radon initiatives for periods of a few years ^2^ ( Réaud *et al.*, 2022).

Following a measurement campaign, the ‘mitigation works’ ( *e.g.* sealing the surfaces of the building in contact with the ground, limiting the transfer of radon in the building, ventilating the basement and/or the living areas) are presented to participating inhabitants. The mitigation is more effective when adequately combined with and adapted to the specificities of the building after a ‘building diagnosis’ performed by an expert ( CEREMA, 2020; Lafage *et al.*, 2017). However, as a result of technical, financial, social and psychological obstacles ( *ibid*.; Bourcier *et al.*, 2010; Hevey, 2017), the number of building diagnoses (even when the expense is covered) and of mitigation works (even in cases when radon concentrations are significant) remains very low ( Nétillard *et al.*, 2013; Turcanu *et al.*, 2020).

Considering that post-measurement steps have been barely addressed by citizen science projects at the time of the development of the protocol and in view of the recurrent difficulties identified in France for this step in the radon management process, **the CEPN chose to focus the pilot project on the building diagnosis phase** performed after the measurement of elevated radon concentrations in houses.

A self-assessment guide for radon in buildings (hereafter called “the guide”) was published on-line in 2019 ^3^. The guide was developed by the Centre for Studies and Expertise on Risks, the Environment, Mobility and Urban Planning (Cerema, a French public agency ^4^), the School of Engineering and Architecture of Fribourg (HEIA-FR ^5^) and the Romand Centre for Indoor Air Quality and Radon (croqAIR ^6^) under the auspice of the JuradBat project ^7^. Designed as a free-to-use interactive questionnaire, the guide provides information to facilitate the understanding of the radon penetration-and-transfer-phenomenon, and proposes specific mitigation solutions that respond to the main characteristics of the building, as indicated by the user.

The pilot project was designed to allow volunteer citizens to use the guide, report on their experience and take part in reflections on its form and the content, thus ensuring that future versions of the guide will be better fit-for-purpose. It is also hoped that the project will increase the visibility of the guide and the adoption of mitigation practices.

In marked contrast to projects with a top-down approach and the clear separation between the citizens and the experts, **the pilot project endeavoured to create the conditions for ‘participatory science’. This meant putting citizens at the core of the project, especially in the production of scientific knowledge and involving them in every different step**, including data collection ( *e.g*. the whole guide is submitted to their analysis), meetings ( *e.g*. the views of the citizens and the experts are treated as equal) and in the overall organization (the development of the project and the conclusions are entirely based on the citizens' answers and proposals ^8^). Therefore, the participants had to be already aware about radon and ideally, had already performed a measurement in their homes and had questions about mitigation—precisely the part of the process the guide is designed for.

To be deemed as ‘citizen science’ and to guarantee the incorporation of recognized standards and best practices in this field, **the project committed to alignment with the ‘Ten Principles’**. While addressing ECSA Principle n°10 (legal and ethical issues surrounding copyright and confidentiality), it seemed necessary to **elaborate a Data Management Plan (DMP)** to delineate which data/results would be open access and which would be kept confidential.

Furthermore, as the ethical grounds of citizen science projects in the field of radon research could be questioned (as experienced by Stanley *et al.*, 2017 and Oberle *et al.*, 2019), **an application form intended for an ethical committee was considered necessary**. An important feature of the ethical considerations was to include in the project protocol the possibility for participants to have a radon diagnosis performed by an expert.

## Methods

### Recruitment of the participants

At the end of 2021, CEPN presented the concept of the pilot project to the Cerema and HEIA-FR experts who developed the guide and invited them to participate. CEPN then contacted the coordinator of the CLS of Pays Vesoul Val-de-Saône (PVVS county ^9^) to discuss the possibility of recruiting participants among the citizens who took part in the winter 2020–2021 radon measurement campaign, where 168 radon measurements were performed in 24 municipalities in PVVS county ( Rivière *et al.*, 2021). In May 2022, approval from the elected representatives of the county was granted and the coordinator sent out a leaflet (Annex 1 of the *Extended data,* Andresz *et al.*, 2023) presenting the project and the terms and conditions to participate, along with the results of the campaign. The researchers expected to recruit around 10% of the persons contacted - a judgement call based on the usual answer rate to survey experienced by the researchers - hence around 15–20 participants, and decided on a minimal value to proceed of 6 participants, which was a bit lower than half the expected value and the size of the groups in Downs *et al.*, 2009; Downs *et al.*, 2010 whose topic of interest was close (air quality). The important consideration in the strategy (an opportunistic sampling) was to recruit a purposive group of motivated individuals to collect information on their experiences and perspectives on a topic of common interest (and not a group sufficient in size to allow statistical analysis of the answers, all the more so since the demographic characteristics of the initial group of 168 participants was not surveyed by PVVS and not known). To this regard, no exclusion criteria were used.

**Figure 1.**
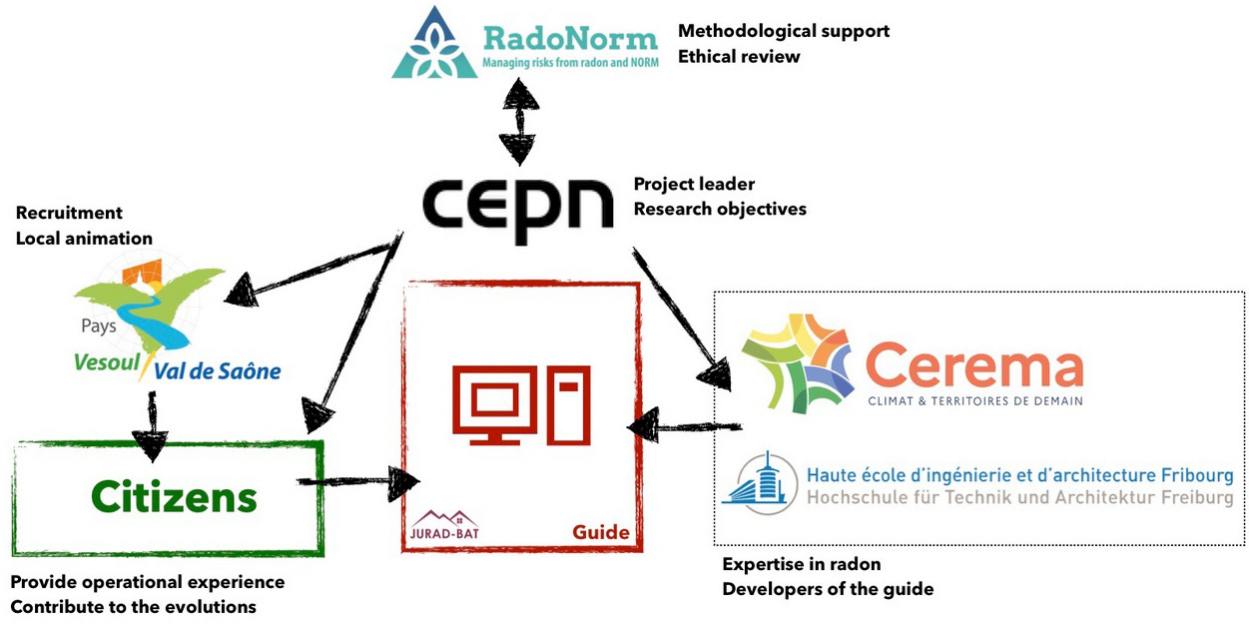
The partners, their relations and the main tasks.

The partners of the pilot project, their relations and main tasks are schematically presented in Figure 1.

### Data collection and analysis

In April-May 2022, the CEPN tested the guide repeatedly and to get accustomed and familiar with the content of the guide and the sequences of the questions depending on the previous answers. A flowchart provided by the developers was also used. The CEPN prepared a questionnaire with the objective to survey all the parts of the guide (the introduction, the questions, the answers provided, the report in .pdf format produced after each use and the section addressing the case where the radon concentration is below 300 Bq.m ^-3^) and from different angles (understanding, usability, suggestions). Due to the exploratory nature of the task, a mixed approach was selected with 24 closed questions, 7 open questions and 5 scaled questions (score from 1 to 5). The questionnaire was tested in-house by the CEPN (for the sake of independence and objectivity, the developers were not involved) and the final version (Annex 2 of the *Extended data,* Andresz *et al.*, 2023) was sent by e-mail to the participants. Quantitative and qualitative analyses of the answers were performed (by CEPN only) to prepare a slideshow for the in-person meetings, whose objective was to provide a forum for citizens and experts to exchange opinions about the guide and discuss possible modifications.

In order to test the guide in real conditions and to further explore its evolution, CEPN decided to compare the methodology established for the guide with that of an independent expert tasked with performing a radon diagnosis in the participants' homes. A protocol to compare the methodologies of the guide and the expert was prepared by CEPN (Annex 3 of the *Extended data,* Andresz *et al.,* 2023) considering all the questions in the guide and evaluating systematically if deviation in these questions and in the final results occur during the on-site diagnosis.

Demonstrating the feasibility of developing a citizen science project for radon management necessarily implies an evaluation of its implementation, the results acquired and the impacts. A feedback questionnaire was prepared on the basis of recommendations made in the evaluation of citizen science projects by RadoNorm partners ( Martell *et al.*, 2022) and in the literature ( Kieslinger *et al.*, 2018; Schaefer *et al.*, 2021 and the dimensions proposed by Phillips *et al.*, 2018). A key feature of the questionnaire (presented in Annex 4 of the *Extended data,* Andresz *et al.,* 2023) is a set of questions addressed to the experts. The value of this expert feedback appears to have been overlooked in earlier projects ( *ibid*.).

### Alignment with the principles of citizen science and the objectives of the project

Prior to its implementation, the above-described protocol was analysed for compliance with the ‘Ten Principles’. The result of this evaluation is presented in Annex 5 of the *Extended data,* Andresz *et al.*, 2023. The consensus among WP6 partners was that the project should be assessed as level 3 'participatory science' on the Hacklay scale ( Table 1).

An ethical application form, inspired by the standard model used by ethics committees in French universities, and a DMP, based on an existing document applicable for Horizon 2020 projects ( CEA, 2021), were elaborated and completed on the basis of the literature review ( Martell *et al.*, 2021), a corpus of recommendations and good practices ( CNIL; Cohen & Doubleday, 2021; Durham, 2012; ECSA, 2020; Pierce & Evram, 2022), and especially those applicable to environment-related health and risk management ( Berti Suman, 2020; Downs *et al.*, 2010; SHAMISEN-SINGS, 2020). The ethical application form and the DMP (Annexes 6 and 7 of the *Extended data,* Andresz *et al*., 2023) were validated by the RadoNorm Ethical Committee 29 August 2022 ^10^. In accordance with these, each participant was given an information letter describing the project and the implications of participation from the outset, and was asked to sign a consent form (Annex 8 of the *Extended data,* Andresz *et al*., 2023). All the participants signed the consent form.

Table 2 presents the connections between the objectives of the pilot project and their operational applications.

**Table 2.** Objectives of the project and materials.

Objectives	Operational applications
Demonstrate the feasibility of a “citizen science” project on radon management in houses	• Check for adherence to the ‘Ten Principles for Citizen Science’ ( Annex 5) • Collect formalized feedback data from citizens and experts about the project for future citizen science initiatives in this area ( Annex 4)
Differentiate from projects focused only on radon awareness and measurement	• Focus on building diagnosis • Use the JuradBat building self-assessment guide for radon • Recruit citizens with prior experience and awareness in radon management ( Annex 1)
Differentiate from the top-down approach and the clear separation between the citizens and the experts	• Put in place the conditions for citizens to be at the very core of the project, involved in all the different steps and using their answers and proposals to guide the project
Obtain results useful for the management of radon and radiation protection	• Produce specifications on the form and the content of the guide to ensure it is better fit-for-purpose • Use questionnaire ( Annex 2), test in real conditions ( Annex 3) and hold meetings • Increase the visibility of the guide
Incorporate ethical considerations	• Prepare an ethical application form ( Annex 6) and a Data Management Plan ( Annex 7) and submit them to the RadoNorm Ethical Committee • Provide an information letter for participants and a consent form ( Annex 8) • Be able to offer a radon building diagnosis performed by an expert to interested participants

## Results

### Implementation

At the end of May 2022, because the number of citizens who had come forward was lower than the minimal value, the leaflet was sent to personnel aware about radon in several local public bodies: PVVS, Cerema, ARS and the Departmental Land Management (DDT). In early June, six participants had been recruited ^11^, and all their questionnaires were collected by mid-June. The analysis of these questionnaires was compiled in a slideshow presented during the first meeting (15 June 2022) and updated before the second meeting (16 June) to take account of the points of view expressed (the second slide show is presented in Annex 9). On 16 June, a building diagnosis was performed (with the help of the blueprints and pictures of the house) for one participant who had requested it and, on 20 July, a diagnosis was performed at the home of another participant (the report is provided in Annex 10).

A remote meeting with the citizens and the experts was planned (22 July 2022) to present and discuss a first version of the results and the final version (see Annex 11) was then sent to all participants. At the beginning of September, feedback questionnaires were sent to the citizens and the experts, and by the end of the month, six questionnaires had been collected.

**Figure 2.**
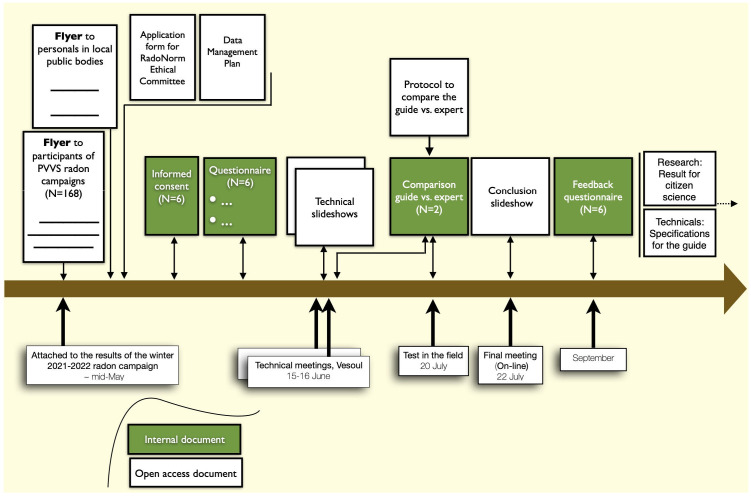
Significant interactions and documents in circulation.

**Figure 3.**
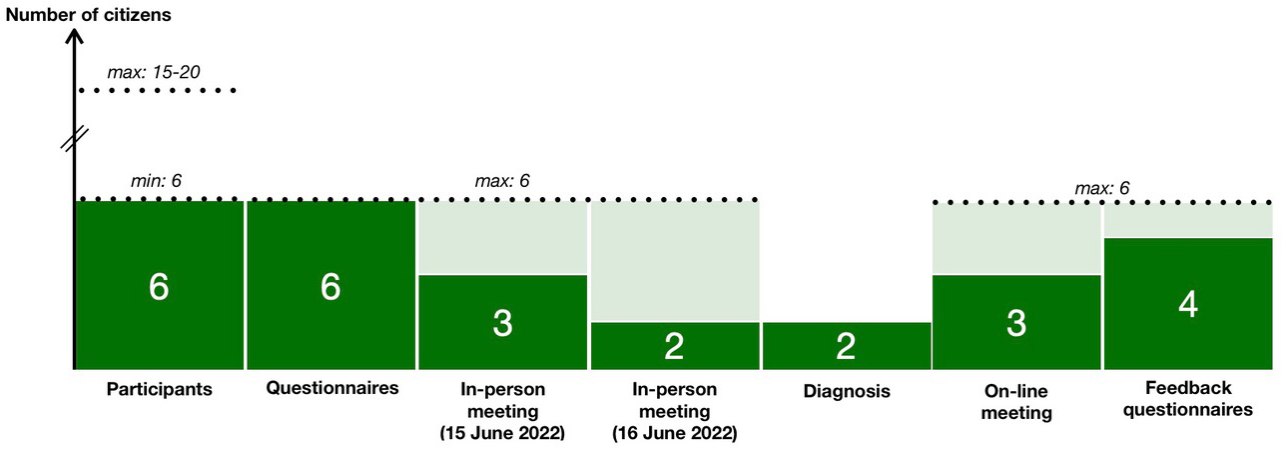
Evolution in the number of citizens in the project steps.

The significant moments of interaction and the documents that have circulated are presented in Figure 2 and the number of citizens in the steps of the project is presented in Figure 3 (the number of experts has always been 2).

### Results of the building self-assessment guide

Analysis of the questionnaires and the discussions during meetings revealed, first and foremost, the recognition by all of the usefulness of the guide and appreciation for its informative and comprehensive nature and the " *enormous amount of work*" it embodies. That being said, several observations and suggestions for improvement were made.

Some suggestions involved the conciseness of the guide. Several parts of the guide were considered too long or detailed:

- The introduction: suggestions were made to split it up and distribute its content in the guide, to record a short introductory video or to design an interactive module using a schematic building to navigate in the guide.
- The pdf report could be shortened by removing repetitions and adding clarity by limiting the number of fonts, replacing line breaks, using colour coding to differentiate between works that are recommended and those to be avoided. No consensus on the ideal size (in pages) of the report was reached.

Other suggestions focused on the need for additional explanations, in particular with regard to ventilation and several technical terms (these were listed). Participants pointed out that technical terms (e.g. “ *ventilation*” or “ *air-tightness defect*”) can be understood very differently by an expert and a citizen. Citizens highlighted that some concepts are better conveyed by image and video (rather than by text), and stressed that these media have become communication standards for the younger (if not for every) generation. All citizens were keen to provide illustrations if needed to replace existing ones.

Finally, whilst the ergonomics of the guide met consensus, some computer bugs and user interface adjustments (such as opening hyperlinks in another tab rather than in the same window) were listed.

Conceptual changes were also suggested by citizens such as incorporating other building configurations than the current ones (or indicating how to use the guide in the case of hybrid building configurations) and using the radon concentration value differently in the diagnosis process. Because the guide was developed by a Franco-Swiss team, it includes some national specificities that proved to be non-transferable between countries (for example: the radon reference level, several building peculiarities, and vocabulary). Participants recommended that the national specificities should be indicated.

The comparison of the guide with the expert confirmed that the guide is by no means a surrogate for the human expert. Only the latter can adapt the diagnosis process to the specificities of the building and decide to use devices (real-time measurement of radon or air renewal) to complete the picture of the radon transfer mechanisms in the house and to apprehend the diverse characteristics of the building (and their interrelationships), resulting in a diagnosis that is unique and inclusive of mitigation tuned to the situation and (possibly) ranked by cost or complexity.

Using the guide and consulting the report contributed to the adoption of diagnosis and mitigation measures by making users more aware of radon risks and how radon penetration/transfer mechanisms work, and by providing a large volume of information about diagnosis and mitigation adapted to broad categories of building configurations, all of which " *makes it possible to anticipate the need for mitigation work*". But several citizens indicated that these elements were neither necessary nor sufficient to act: " *it is imperative to meet a building professional before starting anything*". As the guide serves several objectives, including " *self-assessment*, *education*, *raising awareness*, *explanations of the work*, etc.", some users commented on the technical density of the document and suggested that the number of objectives be limited to the main one, which is (as one developer put it), " *introducing users to the topic of mitigation and to basic skills*".

### Results of the citizen science project

The feedback questionnaires from four citizens (out of six) and two experts (out of two) were analysed. For the citizens, a startling result is that the pilot project had an impact on all tested aspects:

• The project raised citizen awareness about (a) radon risks in houses, (b) building diagnosis and (c) mitigation (impact on the *interest* dimension under Philips *et al.* typology (Philips, 2018));
• It increased the level of information citizens have on these 3 topics ( *knowledge*);
• It encouraged citizens to look for further information ( *skills of science inquiry*);
• It enabled citizens to talk about these 3 topics ( *stewardship*);
• It motivated citizens to take actions ( *self-efficacy*), to change their habits ( *behaviour*) both at home and in their circle of family, friends, and neighbours.

The open comments emphasized the “ *rewarding*” nature of the project, allowing a large volume of information to circulate from the experts to the citizens. It was observed that the citizen science approach allowed people to “ *digest*” the information better.

For their part, the experts reported an improved awareness about citizen science applied to radon management efforts. They commented on their improved understanding of several knowledge and perception gaps between experts and citizens (" *the difficulty for individuals* [...] *to understand the issues*") and that other forms of support for diagnosis and mitigation are needed for action to occur after radon measurement. While the project did not change their technical knowledge about diagnosis and mitigation, “ *the way of addressing these subjects*” in the future will be optimized by adapting the message and providing more room for explanations and pedagogy. Finally, the experts concluded that they were willing to talk about the pilot project in their organisations and beyond, via their networks ^12^.

## Discussions

### Project design and protocol

Citizen science is a flexible term and different metrics are available to design a ‘citizen science’ project be they quality criteria ( Heigl & Dörler, 2018), best practices ( EU, 2020) or research ethics board recommendations ( Oberle *et al*., 2019), although their applicability will depend among others: on the national regulations (on radon, ethics, data management), the targeted population, the objectives of the project and who is funding. In the context of the European RadoNorm project and previous publication of the partners ( Martell *et al*., 2021) choosing the ECSA 10 principles as indicators for the elaboration of the pilot project was the logical option.

At the design stage, the pilot project was confronted with the absence of a framework concerning the inclusion of ethical considerations for a citizen science project on radon: the literature review found that an ethics committee was not a systematic feature ( *ibid.*, that its conclusions can be questioned ( Oberle *et al.*, 2019) and that the content of the standard existing ethical application form did not align with the challenges of citizen science and radiation protection ^13^. For the pilot project, it appeared necessary to adapt an existing file (for details about the adaptation, see Annex 7).

If citizen science on radon were to develop, the pilot projects would benefit from a harmonized set of recommendations to decide whether approval from an ethics committee is needed. A template integrating the ethical principles of citizen science ( *e.g*. ECSA, 2020) and possibly the ethical principles and procedural values of radiation protection ( ICRP, 2018) would also be helpful in this respect.

Principle 7 ( ECSA, 2015) invites project leaders to make data and results publicly available and to publish in Open Access and in citizen science databases, but this may conflict with the need to respect the confidentiality of the participants, who run the risk of facing a potential devaluation of their property or being targeted by radon solution commercial providers. In previous citizen science projects on radon, the results were anonymized ( Tsapalov *et al.*, 2020).

For the pilot project, the following practices were integrated in the DMP ( Annex 7) and these could form the basis for the data management of future citizen science projects applied to radon management in houses:

• A data protection impact assessment, expressing the risk/benefit balance for participating citizens;
• Integrating recommendations from citizen science practitioners ( Cohen & Doubleday, 2021; Durham, 2012; Pierce & Evram, 2022) and the French National Commission for Data Protection and Liberties ‘Six Key Practices’ ( CNIL);
• Consideration of the FAIR principles ( EU, 2016);
• An informed consent to participate ( Annex 8) describing how confidentiality is guaranteed and giving the possibility for participants to contact researchers at any time.

The researchers have endeavor to meet all ECSA 10 Principles in the pilot-project. However, their application from the onset on the management of radon in home proved to be challenging: some principles need adaptation, thematic could be missing and also applying all principles might be erring on the side of caution and potentially distracting resources and time from other benefiting goals of the project (recruitment, outcomes ...). The results of the other WP6 RadoNorm pilot-projects might confirm (or not) if applying all 10 Principles is a viable decision for citizen-science projects associated with radon management.

### Participation

Citizen participation was an aspect that received constant attention. At the beginning, the challenge lays in the constitution of a group of sufficient size to generate discussions with the experts and to bring together different points of view (N.B. the characteristics of the population who participated in the radon measurement campaign were not known, so representativeness was not sought). Although the number of citizens was limited - which was considered “ *most unfortunate*” by all – this count is not uncommon ( Downs *et al.*, 2009; Downs *et al.*, 2010) and was ultimately quite convenient as it allowed for rich and inclusive discussions that are not necessarily possible with a large group.

Maintaining the level of participation throughout the project was another challenge ( Figure 3). To counteract the potential for ‘participation fatigue’, the pilot project was concentrated in a short time frame (< 5 months) with meetings planned outside weekends and working hours (18:00 to 21:00). Regular follow-ups and reminders by email and telephone were sent out by CEPN and the CLS coordinator. Yet, if all the participants returned the questionnaire, only half of them attended the meetings (probably due to the inconvenience of the distance) and six feedback questionnaires (out of eight) were collected.

The researchers acknowledged that the participation during the project has been limited, especially in comparison with some projects identified in the literature review ( Martell *et al*., 2021) and recent Canadian projects gathering between two to four thousand participants (ex. Cholowsky *et al*., 2023; Irvine *et al.*, 2022). It has however to be kept in mind that this pilot project was a prototype to test the feasibility of applying citizen science to improve radon diagnosis and remediation, and not meant to reach a broad scale. Among the possible (combining) reasons that explain the small participation: an opportunistic targeted sampling, an initial target of small size, a small team of researchers, the erosion of the interest of the inhabitants about radon (the measurement took place in winter 2020–2021), the complexity of the topic as well as social factors and behavioral reactions towards radon.

The motivations driving participants to join the project were surveyed orally. The " *European*" nature of the project, which gives it “ *quite a dimension*” and “ *appeal*” (because opportunities to participate in this type of project are rare) was highlighted. Citizens also indicated that the topic itself was " *interesting*" and " *important*". All these motivations relate to the concept of 'collective motivation' (under the classification established by Nov *et al.*, 2011 and recommended by Martell *et al.*, 2022). Several participants were also looking for information about diagnosis and mitigation (" *I wanted to know more*"), which corresponds to the concept of ‘extrinsic motivation’. These are the two forms of motivation that were explicitly expressed.

To generate and sustain participation in future radon-focused citizen science projects, several scenarios could be explored in different combinations, depending on the topic:

• Emphasize the collective and extrinsic motivations identified in the pilot project;
• Discuss activating other forms of motivation, *e.g.* social interaction, reputation, and norm-oriented behaviour (see Nov *et al.*, 2011), but bearing in mind that the financial compensation of citizens (an intrinsic motive) is not an insignificant decision and could create difficulties ( Tauginiene *et al.*, 2021);
• Consider the geographical extension of the project, but a large-scale-project does not facilitate plenary in-person meetings and remote meetings have their own challenges ( Fouqueray *et al.*, 2023);
• Adapt the scope of the project in terms of the number and type of participants, for example by involving citizens who have not previously performed radon measurements (in which case, mass media can be used including printed media, municipal newsletters, social networks, microinfluencers ( Cholowsky *et al*., 2023) etc.);
• Mobilize pre-existing and well-established scientific organizations, ideally with experience in citizen science, *e.g*. a citizen laboratory. Platforms used for sharing citizen science projects (e.g. eu-citizen.science, scistarter.org) can be screened;
• Align the project's timetable with local initiatives ( *e.g.* coordinate the start the project with a radon measurement campaign).

### Results

The project generated a significant volume of comments and suggestions which, in the opinion of the experts, were " *constructive*", " *interesting*", of high quality and constituting a pool of modifications for the guide. While some suggestions can be implemented easily in the short term, others require a longer process of development and arbitration. It should be noted that at the time of writing, the transfer of the administration of the Jurad-Bat platform - which hosts the guide - to a new body was under negotiation. This transition is likely to delay the integration of the modifications proposed by citizens but will also strengthen the robustness and sustainability of the platform.

The results were collected through questionnaires and in-person/online meetings, which are quite 'classical' means for experts. For future citizen science projects (and if applicable), digital-methods including video, social media or smartphone applications could be implemented (the latter has already been applied in citizen science on ionizing radiation, Kim *et al.*, 2020; SHAMISEN-SINGS, 2020; Tsapalov *et al.*, 2020). The raw responses to the questionnaires can be found as *Underlying data* ( Andresz *et al.*, 2023).

### Merging citizen science and radon management

Citizen science initiatives are a way to federate citizens on the development of comprehensive and participatory approaches to radon risk management and strengthen their engagement in post-measurement actions, both being essential to fostering the radon management process ( Turcanu *et al.*, 2020). However, the pilot project has shed light on some specific difficulties when citizen science meets radon.

Firstly, it is difficult to awaken the interest of citizens in radon: a complex, little-known, yet worrying subject that requires prior knowledge and familiarity with specific vocabulary and that may lack appeal in relation to other subjects. Other projects were much efficient in raising awareness yet after several interactions (2–5 over a year in general, or more interactions, Cholowsky *et al.*, 2023; Irvine *et al.*, 2022).

Secondly, as conceptualized for environment-related issues ( Berti Suman, 2020; Ceccaroni *et al.*, 2021), citizen science focusing on a given health risk (be it air or soil pollution, invasive species, etc.) must be able to provide support for the management of the risk 'revealed' to participants. As a consequence, a citizen science project involving individuals exposed to radon in their homes should provide the (human) expertise, time and budget necessary to manage the risk. However, in France, this expertise is scarce, the effectiveness of the mitigation works is hard to predict and their costs difficult to determine. These issues should be considered by researchers when designing and framing future citizen science projects on radon management in houses.

A citizen science project should produce " *a genuine science outcome*" (in accordance with Principle 2 of ECSA, 2015), yet the implementation of the diagnosis and mitigation is based on know-how and a portion of empiricism. This observation does not impair their efficiency, yet there remains some level of interpretation on whether a citizen science project aiming to improve diagnosis and mitigation measures is 'scientific' or not. Without trying to discourage new projects from tackling the issues that arise post-measurement, the importance of correctly ascertaining the scientific aspects of the project should not be overlooked.

Beyond these difficulties, the participants in the pilot projects indicated that citizen science also brings new perspectives on ways to improve the management of radon. The following items were identified:

• Include a citizen science project in a Local Health Contract;
• Encourage established networks with an interest in indoor air quality and radon to start a citizen science project;
• Take advantage of the firm connections with actors in the field to improve the implementation of mitigation solutions and to collect feedback on their efficiency and cost (which are very rare data);
• Collect information about the expectations/difficulties faced by citizens;
• Introduce new ways to make people more aware and better trained in the area of radon management, contributing to (more) informed decision-making;
• Enlarge the circle of reflection beyond the experts.

## Conclusions

### Synthesis of the results

This experience demonstrates that it is possible to design a project that meets the established and recognized principles of citizen science, can be deemed ‘participatory science’, and whose focal point is the building diagnosis that follows a radon measurement at home. High standards for data management and considerations for ethical principles were adopted and adapted to the specificities of the project. All these elements constitute notable methodological outcomes.

The technical results are rich and constructive. They will nurture modifications and developments to the guide at short and medium terms. The project confirmed the usefulness of the guide, which contributes to fostering action in the radon post-measurement phase. The project also increased the visibility of the guide and enlarged its initial audience.

Citizen participation was limited, but the project, being a pilot, was not designed to reach a large scale and it was not a barrier as the small group produced rich and inclusive discussions. Evaluation of the feedback highlights the benefits of the project for citizens in terms of the information received, how actionable this information can be, and how the citizens will disseminate it further and ultimately change behaviours in a wider population.

As for the experts (whose feedback is rarely collected after a citizen science project), this project has sharpened their awareness about the gaps that may exist between themselves and the citizens. Experts were thus enabled to adapt their approaches to the subject.

The pilot project encountered several specific issues that arise when citizen science meets radon, but in the end, these were found to be manageable. Both the citizens and the experts maintained that the citizen science approach has great unexplored potential to strengthen radon management at individual, local and potentially even bigger scales.

### Perspectives

This pilot project is one of the four projects in the ‘incubator of citizen science models’ set up in the framework of RadoNorm WP6 which, at the time of writing, were being implemented in Ireland, Norway and Hungary. Each pilot project has different objectives and its own modalities ( Martell *et al.*, 2022). In 2023, WP6 moved into the second phase by supporting organizations willing to carry out citizen science projects on radon management in houses. The organizations were selected after an open call which run from November 2022 to February 2023. In a third and final phase (post 2023), "citizen science toolkits" will be constituted by the RadoNorm partners on the basis of the experiences and elements – methodological, technical, tools, recommendations – identified in the projects. These toolkits will be made available to any actors wishing to set up a citizen science project applied to radon management in houses.

### Acronyms

**ARS**: Regional Health Agency; **CEPN**: Nuclear Protection Evaluation Centre; **Cerema**: Centre for Studies and Expertise on Risks, the Environment, Mobility and Urban Planning; **CLS**: Local Health Contract; **DDT**: Department for Land Management; **ECSA**: European Citizen Science Association; **DMP**: Data Management Plan (Annex 7); **HEIA-FR**: School of Engineering and Architecture – Fribourg; **PPVS**: Pays Vesoul Val-de-Saône; **WP**: Work Package.

## Data Availability

Store ^DB^: STOREDB:STUDY1177 RadoNorm Subtask 6.3.1 - Citizen science pilot-project - Application in France - Documents - 2021~2022. https://doi.org/10.20348/STOREDB/1177 ( Andresz *et al*., 2023). This project contains the following underlying data: -   Underlying data RadoNorm WP 6 3 1 Citizen Science French pilot project.xlsx Tab 'Questionnaire': response to the questionnaires (see Annexe 2 of the Extended data) Tab 'Feedback questionnaire': responses to the feedback questionnaire (see Annexe 4 of the Extended data) -   [Raw questionnaires associated with] Underlying Data RadoNorm WP 6 3 1 Citizen Science French pilot project.pdf (raw responses in French) Store ^DB^: STOREDB:STUDY1177 RadoNorm Subtask 6.3.1 - Citizen science pilot-project - Application in France - Documents - 2021~2022. https://doi.org/10.20348/STOREDB/1177 ( Andresz *et al.*, 2023). This project contains the following extended data -   Annexe 1 — Leaflet for the recruitment of participants (in French) -   Annexe 2 — Questionnaire about the building self-assessment guide -   Annexe 3 – Protocol to compare the building self-evaluation guide with an expert -   Annexe 4 — Feedback questionnaire about the participation to the pilot-project -   Annexe 5 – Adequation of the pilot-project with the ten principles in citizen science -   Annexe 6 — Application form for ethical committee and data management plan -   Annexe 7 — Data management plan -   Annexe 8 — Information document and consent form -   Annexe 9 —Slideshow prepared for and during the in person-meetings, 15 and 16 June 2022 (in French) -   Annexe 10 – Radon expertise report from the visit performed 20 July 2022 (Cerema document) (in French) -   Annexe 11 — Slideshow prepared for and validated after the final meeting, 22 July 2022 (in French) Data are available under the terms of the Creative Commons Attribution 4.0 International license (CC-BY 4.0).
