# Supplementary material for: When citizen science meets radon building diagnosis: Synthesis of a French pilot project developed in the framework of the European RadoNorm research project
Source: Open Res Eur. 2023 Aug 8;3:106. Originally published 2023 Jun 22. [Version 2] doi: 10.12688/openreseurope.15968.2 (PMC10511845; doi:10.12688/openreseurope.15968.2)
Supplement: Peer review history

# Reviewer report: Reviewer response for version 2

**DOI:** 10.21956/openreseurope.17763.r34335

**Published:** 2023-09-20

**Authors:** Aaron Goodarzi

**Affiliations:** 1 Robson DNA Science Centre, Department of Biochemistry and Molecular Biology, Charbonneau Cancer Institute, Cumming School of Medicine, University of Calgary, Calgary, Alberta, Canada

Having read the revisions, I am satisfied that this report is sufficiently well-described in terms of what was done and the outcomes that it is suitable for the scientific community to evaluate on it's merits, and learn from as progress is made within the space of increasing radon awareness and reducing high exposures. 

 No further comments or suggestions.

If applicable, is the statistical analysis and its interpretation appropriate?

Not applicable

Is the study design appropriate and is the work technically sound?

Partly

Is the work clearly and accurately presented and does it engage with the current literature?

Partly

Are the conclusions drawn adequately supported by the results?

Partly

Are sufficient details of methods and analysis provided to allow replication by others?

Yes

Are all the source data and materials underlying the results available?

Yes

Reviewer Expertise:

Citizen science, radon exposure, radiation exposure, population health, radiobiology, building science

I confirm that I have read this submission and believe that I have an appropriate level of expertise to confirm that it is of an acceptable scientific standard.

# Reviewer report: Reviewer response for version 2

**DOI:** 10.21956/openreseurope.17763.r34334

**Published:** 2023-08-08

**Authors:** Boris Dehandschutter

**Affiliations:** 1 Federal Agency for Nuclear Control, Brussels, Belgium

Thank you for the revision, I have no further comments to make.

If applicable, is the statistical analysis and its interpretation appropriate?

Not applicable

Is the study design appropriate and is the work technically sound?

Yes

Is the work clearly and accurately presented and does it engage with the current literature?

Yes

Are the conclusions drawn adequately supported by the results?

Yes

Are sufficient details of methods and analysis provided to allow replication by others?

Yes

Are all the source data and materials underlying the results available?

Yes

Reviewer Expertise:

Environmental radioactivity expert at the Federal Agency for nuclear control, the radiation protection authority in Belgium

I confirm that I have read this submission and believe that I have an appropriate level of expertise to confirm that it is of an acceptable scientific standard.

# Reviewer report: Reviewer response for version 1

**DOI:** 10.21956/openreseurope.17242.r33200

**Published:** 2023-07-20

**Authors:** Aaron Goodarzi

**Affiliations:** 1 Robson DNA Science Centre, Department of Biochemistry and Molecular Biology, Charbonneau Cancer Institute, Cumming School of Medicine, University of Calgary, Calgary, Alberta, Canada

This study involves exploring the feasibility of carrying out a citizen science style radon awareness project in a French community. The study team attempt to apply all ten principles of citizen science into their research, which is an ambitious goal. While the study team have clearly learned a variety of valuable lessons regarding the implementation of a radon awareness project via a citizen science approach, regrettably the lack of participation by the community (6 responses) does detract from the outcomes. In my view, this lack of participation is, itself, a primary outcome that is indicative that the approach used here is problematic. I would like to see a more fulsome discussion of this, as it is valuable for others to understand. For example, I think a clear lesson from this work is that trying to apply all ten (academic) principles of citizen science (while a laudable goal) is now demonstrably not practical for this type of work, and appears to have interfered with the teams ultimate goal of promoting broad scale radon awareness, increasing understanding of local buildings as they relate to radon, and motivating health-seeking behaviour such as radon testing and exposure reduction. 

 This work could be improved by re-framing some of the discussion in terms of compare-and-contrast with other global jurisdictions where citizen science style work in radon awareness and exposure reduction are ongoing (e.g. Canada). I would recommend the authors be open to more boldly stating whether excluding certain principles of citizen science might be beneficial. 

 Within the discussion section, I would challenge the notion that it is universally "...difficult to awaken the interest of citizens in radon...". While this has been the experience of this team, there are now a variety of studies elsewhere that provide evidence that awakening this interest is achievable. Recent publication from citizen science centric research projects in Canada involve >30,000 households and achieve broad scale notoriety in the Canadian national press. While certainly there are many differences between Canada and France, the Canadian example is a valuable point of reference against which this can be compared to derive lessons.

**Minor comments:**

 Reference "Fintan *et al*., 2017" is incorrectly labelled, as this should be Stanley *et al*., 2017 (Dr. Fintan Stanley being the first author). 

 Some additional references would be useful within the introduction, such as primary literature citations regarding exposure doses linked with increased lung cancer risk. I would also say it would be appropriate to provide a quick update on the information presented in Martell *et al*., 2021, as many additional studies involving citizen science and radon have emerged since then, making the statement that "number of papers published on citizen science projects applied to radon management and research has been modest: less than 10 projects over the last decades" out of date / untrue as of this time. 

 It is also incorrect to say that previous citizen science studies "focused only on the first steps of radon management – namely, information about radon and measurement at home – while the post-measurement stages focusing on reducing radon exposure were not tackled." There have been at least three studies since 2021 that addressed post measurement stages, citizen scientist psychology, reactions to information, behavioural choices, and more. So this section within the introduction would benefit from an update. Relevant PMID for these and other citizen science based radon publications include: 37029226 ^1^, 36104382 ^2^, 34475435 ^3^, 34099826 ^4^, 33762674 ^5^.

If applicable, is the statistical analysis and its interpretation appropriate?

Not applicable

Is the study design appropriate and is the work technically sound?

Partly

Is the work clearly and accurately presented and does it engage with the current literature?

Partly

Are the conclusions drawn adequately supported by the results?

Partly

Are sufficient details of methods and analysis provided to allow replication by others?

Yes

Are all the source data and materials underlying the results available?

Yes

Reviewer Expertise:

Citizen science, radon exposure, radiation exposure, population health, radiobiology, building science

I confirm that I have read this submission and believe that I have an appropriate level of expertise to confirm that it is of an acceptable scientific standard, however I have significant reservations, as outlined above.

## References

[1] : Consequences of changing Canadian activity patterns since the COVID-19 pandemic include increased residential radon gas exposure for younger people. *Sci Rep* .2023;13(1) : 10.1038/s41598-023-32416-8 5735 10.1038/s41598-023-32416-8 37029226PMC10081328

[2] : Social factors and behavioural reactions to radon test outcomes underlie differences in radiation exposure dose, independent of household radon level. *Sci Rep* .2022;12(1) : 10.1038/s41598-022-19499-5 15471 10.1038/s41598-022-19499-5 36104382PMC9473468

[3] : Rising Canadian and falling Swedish radon gas exposure as a consequence of 20th to 21st century residential build practices. *Sci Rep* .2021;11(1) : 10.1038/s41598-021-96928-x 17551 10.1038/s41598-021-96928-x 34475435PMC8413302

[4] : The efficacy of public health information for encouraging radon gas awareness and testing varies by audience age, sex and profession. *Sci Rep* .2021;11(1) : 10.1038/s41598-021-91479-7 11906 10.1038/s41598-021-91479-7 34099826PMC8185097

[5] : Younger North Americans are exposed to more radon gas due to occupancy biases within the residential built environment. *Sci Rep* .2021;11(1) : 10.1038/s41598-021-86096-3 6724 10.1038/s41598-021-86096-3 33762674PMC7990966

# Response

**Published:** 2023-07-28

**Authors:** Sylvain Andresz

**Affiliations:** 92260, Nuclear Protection Evaluation Centre (CEPN), Fontenay-aux-Roses, Île de France, France

Dear Professor Goodarzi, Thank you very much for your review, positive feedback and helpful comments. We have implemented your comments (as well as elements coming from the comments until 24 July and further proof-checks) in a new version. In the following text, we would like to answer your comments in order of appearance in your report. 

 “ *I would like to see a more fulsome discussion of this [the low participation of the citizen to the project], as it is valuable for others to understand*”.

- We have included a § in the ‘Participation’ plaining detailing the possible reasons why the participation was low in the end.

“ *For example, I think a clear lesson from this work is that trying to apply all ten (academic) principles of citizen science (while a laudable goal) is now demonstrably not practical for this type of work, and appears to have interfered with the teams ultimate goal of promoting broad scale radon awareness, increasing understanding of local buildings as they relate to radon, and motivating health-seeking behaviour such as radon testing and exposure reduction*” […] *I would recommend the authors be open to more boldly stating whether excluding certain principles of citizen science might be beneficial*”.

- The 10 Principles were the metrics used by the WP6 Leaders and Partners to assess whether a project can be regarded as citizen science or not and (maybe erring on the side of caution) we decided to try to meet all the 10 Principles. It can be argued that this decision might have distracted resources/time from other aspects of the projects – maybe more beneficial. However, the Principles are a list of good practices, published almsot a decade ago, coming from the field and aiming to support the design and implementation of citizen science projects. It is not clear to the researchers which Principle(s) could have interfered (on a conceptual level) with the pilot project.
- The outcomes of the pilot project are limited anyway (this was prototype, limited in size, …) and we are unsure they can be utilized to confront the 10 Principles. We then propose to see if the other RadoNorm pilot-projects also experienced that the application of the 10 Principles was not a viable option.
- We have added a § detailing these reasons in the ‘Project Design and Protocol’. This was also the opportunity to indicate the existence of other metrics to decide if a project is CS and open a discussion on differences between countries (a comment of yours).

“ *I would challenge the notion that it is universally "...difficult to awaken the interest of citizens in radon...*".

- Following the recent publications (not captured by the initial literature review), this statement is no longer true indeed. We have adjusted the statement by including results of 2 of the articles (among those with the highest citizen participation) you recommended.

“ *Some additional references would be useful within the introduction, such as primary literature citations regarding exposure doses linked with increased lung cancer risk*.”

- Inclusion of the "classical" publications of Darby and colleagues and Krewski and colleagues on the epidemiological results showing the dose-response relationship in the Introduction. Thanks for the recommendations.

“ *I would also say it would be appropriate to provide a quick update on the information presented in Martell et al., 2021 making the statement that "number of papers published on citizen science projects applied to radon management and research has been modest: less than 10 projects over the last decades" out of date / untrue as of this time.  […] It is also incorrect to say that previous citizen science studies "focused only on the first steps of radon management*”. 

- The literature analysis performed by the Leaders and Partners of the RadoNorm WP6 project (the reference Martell et al. 2021) seek publications from 1984 to October 2020 (therefore could not capture later publications) and was published in June 2021 (at the very moment the team started the redaction of the protocol and make contact with the French experts).
- A new § has been included in the ‘State of the art’ part to indicate the existence and scope of the recent publications and experiences from Canada. Also, in the ‘Objectives’, we indicated that post-measurement steps were barely addressed by citizen science projects *at the time of the development of the protocol*.

Thanks again for your revision and comments. Sylvain Andresz, on behalf of the co-authors.

# Reviewer report: Reviewer response for version 1

**DOI:** 10.21956/openreseurope.17242.r33199

**Published:** 2023-07-13

**Authors:** Boris Dehandschutter

**Affiliations:** 1 Federal Agency for Nuclear Control, Brussels, Belgium

The paper describes a pilot project of citizen science for radon management, where the authors attempted to recruit citizens in order to apply a bottom-up approach to radon management in dwellings, since this topic is of particular importance as radon affects peoples personal environment (their houses). Although it turned out to be very difficult to recruit sufficient candidates, because of the complexity of the topic, the amount of time needed to investigate, the paper can be of interest and importance for future research and attempts to imply citizen science in radon management programs. The context, framework and scientific basis on the citizen science approach are well elaborated, as is the overall methodology. The conclusions are rather limited, due to the limited number of citizen candidates recruited. This problem is well known and addressed in the paper in the section 'Participation' of the discussion. This pilot study provides a good basis for further initiatives and research for increasing public involvement in the radon programmes, and for using the input from those directly affected (the citizens) in their development and improvement.

If applicable, is the statistical analysis and its interpretation appropriate?

Not applicable

Is the study design appropriate and is the work technically sound?

Yes

Is the work clearly and accurately presented and does it engage with the current literature?

Yes

Are the conclusions drawn adequately supported by the results?

Yes

Are sufficient details of methods and analysis provided to allow replication by others?

Yes

Are all the source data and materials underlying the results available?

Yes

Reviewer Expertise:

Environmental radioactivity expert at the Federal Agency for nuclear control, the radiation protection authority in Belgium

I confirm that I have read this submission and believe that I have an appropriate level of expertise to confirm that it is of an acceptable scientific standard.

# Response

**Published:** 2023-07-28

**Authors:** Sylvain Andresz

**Affiliations:** 92260, Nuclear Protection Evaluation Centre (CEPN), Fontenay-aux-Roses, Île de France, France

Dear Mr. Dehandshutter, Thank you very much for your review and approval. Sylvain Andresz, on behalf of the authors.
